# Postnatal interventions to support newborn survival: updates for the Lives Saved Tool

**DOI:** 10.7189/jogh.16.04272

**Published:** 2026-10-02

**Authors:** Tyler Vaivada, Yvonne Tam, Georgia Dominguez, Li Jiang, Rachel Lee Him, Oviya Muralidharan, Davneet Sihota, Leila Harrison, Robert E Black, Zulfiqar A Bhutta

**Affiliations:** 1 Center for Global Child Health, Hospital for Sick Children, Toronto, Canada; 2 Department of International Health, Bloomberg School of Public Health, Johns Hopkins University, Baltimore, USA; 3 Institute for Global Health and Development, The Aga Khan University, Karachi, Pakistan

**Keywords:** neonate, newborn care, neonatal mortality, postnatal, interventions, Lives Saved Tool (LiST)

## Abstract

**Background:**

The period immediately after birth represents a highly vulnerable time for newborns, particularly in contexts without access to high-quality secondary care for small and/or sick newborns. Scaling up existing evidence-based interventions can avert much of the mortality and morbidity that occurs during the neonatal period. We summarise the evidence for interventions delivered postnatally and propose a set of effect estimates for inclusion in the Lives Saved Tool (LiST).

**Methods:**

We first conducted a set of systematic reviews and updates on the most recent evidence from randomised controlled trials on the effectiveness of interventions for reducing neonatal mortality and associated risk factors. We then assessed the evidence using standardised set of considerations alongside an iterative consultation process with newborn care experts to determine whether and how new meta-analysed effect estimates could be incorporated into the LiST model, as well as which of these estimates should be updated and which should be retained.

**Results:**

We proposed a new set of interventions that involve three packages of newborn care: a basic package (*i.e.* primary/community, secondary hospital care), a referral care package (*i.e.* tertiary care), and a package of promising innovative interventions whose evidence base is still developing. We included five new interventions within the updated LiST model’s basic and referral packages: nasal continuous positive airway pressure coupled with preterm supportive care, newborn referral and transportation, animal-derived surfactant, prophylactic antifungals, and antibiotic stewardship. Three new interventions were proposed for the innovation package: magnesium sulphate for neuroprotection in asphyxiated newborns, topical emollient therapy, and probiotics/synbiotics for hospitalised preterm newborns.

**Conclusions:**

We synthesised the evidence base for the impact of a package of effective postnatal interventions for neonates for an updated LiST model. The new interventions and updated effect estimates can provide more accurate projections of potential impacts on neonatal survival for different intervention coverage scale-up scenarios in low- and middle-income countries.

Despite some reductions over the past decades, rates of stillbirths and neonatal mortality remain high in many low- and middle-income countries (LMICs), with an estimated two million stillbirths and 2.28 million neonatal deaths in 2023 [1]. Even in the context of an increasing proportion of mothers delivering within health facilities, corresponding decreases in neonatal mortality and morbidity have not been as rapid as expected, perhaps due to variability in the availability and quality of antenatal and postnatal care. A variety of specific evidence-based interventions that are part of larger packages of care have been found to effectively reduce the risk of stillbirths and neonatal death [2] through reductions in risks related to the main causes of neonatal mortality worldwide: preterm birth complications, birth asphyxia and intrapartum complications, congenital anomalies, and neonatal infections [3].

We build on a recent body of work that has sought to update the evidence synthesis literature on the effectiveness of maternal and newborn interventions, with a particular focus on facility-based interventions at higher levels of care (*i.e.* secondary and tertiary) [4–10]. This is due to increases in coverage of facility births and access to neonatal care, concerns with maternal and neonatal care quality, and the large number of preventable neonatal deaths that occur in facilities. To this end, we published a series of systematic reviews [4] as a successor to the 2014 Every Newborn Series and associated Every Newborn Action Plan [2], and herein describe the process of assessing the potential impact of these new intervention packages across the continuum of maternal and newborn care using the established Lives Saved Tool (LiST), which has been used extensively to estimate impact on maternal and newborn deaths and other outcomes [2,11].

The LiST is a publicly available application designed for researchers, programme planners, policy makers, and other stakeholders to help model and identify the optimal mix of evidence-based interventions to reduce rates of neonatal and child mortality in LMICs with varying levels of intervention coverage [11]. Since many existing effect estimates in the LiST come from systematic reviews and other publications that are more than ten years old, we sought to collate updated estimates for inclusion in LiST to improve the accuracy of lives saved predictions using the best available evidence. We derived the specific effect sizes associated with the scale-up of each intervention within LiST from pooled effect estimates reported in meta-analyses and robust systematic reviews of intervention effectiveness, and from Delphi approaches where this was not feasible because of ethical considerations or a lack of data from high-quality experimental studies.

In this modelling input paper, we document the specific sources and derivation of pooled effect estimates for interventions that have contributed to the updated LiST model and provide rationale for inclusion decisions. We report only on the set of interventions that are delivered immediately after childbirth and into the postnatal period, while a complementary paper examines the evidence for antenatal and intrapartum care interventions [12].

## METHODS

We previously conducted an initial intervention scoping exercise and a series of overviews of reviews, new systematic reviews, review updates, and re-analyses of LMIC-specific data between 2022 and 2024 [4,13] to identify the most robust evidence for antenatal and neonatal care interventions. We prioritised studies conducted in low-income, lower-middle-income, and upper-middle-income countries (based on the World Bank classification [14]). When evidence from LMICs was insufficient, we leveraged evidence from high-income countries (HICs). The methodology for this review process is detailed elsewhere [4], while specific methods are described in the individual postnatal intervention-specific papers [5–10].

Among the reviewed interventions, we identified a subset as candidates for inclusion in the LiST model through a series of consultations with a technical advisory group (TAG) and the team at Johns Hopkins University (YT and REB) overseeing the LiST technical model and applications. The process and guidelines for selecting intervention effects for use within LiST have been described previously [15]. In short, the primary requirement was that the effect of the postnatal intervention on neonatal mortality be reported as a pooled estimate, with priority given to cause-specific mortality outcomes when available. When only all-cause mortality was reported, the primary underlying cause(s) of death for the condition targeted by the intervention were assigned as the cause of neonatal mortality within LiST.

For certain interventions such as injectable antibiotics, trials could not be designed with true control groups with no treatment, given that it is unethical to withhold antibiotics from hospitalised newborns with possible severe bacterial infections. We pooled survival rates after injectable antibiotic treatment across LMIC trials. To simplify comparison, we used a counterfactual of 100% mortality in these cases, as it is assumed these newborns would have been unlikely to survive before modern antimicrobial therapy.

The original set of interventions already included in the LiST model represented the foundation for this work [2]; revising their existing estimates (where applicable) using more up-to-date literature was a primary focus. We also attempted to provide further granularity to existing neonatal ‘packages’ in the LiST that contained more than one intervention, but were previously modelled in aggregate. This included the packages for full preterm supportive care and full supportive care for neonatal sepsis/pneumonia. We also identified several new interventions already delivered as part of small or sick newborn care, but whose effectiveness had not previously been quantified for inclusion in the LiST.

We organised individual interventions by critical window groups to highlight when they should be delivered to maximise impact. We organised these individual interventions into three distinct packages according to the types of health facilities in which they are delivered: basic, referral, or innovation. The LiST models the individual intervention effects along with other individual interventions, and ‘packages’ are the aggregate effects of the interventions that were designated to be in each package. Users can choose to assess the impact of individual interventions, multiple interventions, or a full package of interventions.

We reported the effect estimates, 95% confidence intervals (CIs), quality assessments, and other relevant details from new, updated, re-analysed, and existing meta-analyses. We also included an assessment of the overall quality of the evidence for each intervention’s effect on mortality outcomes using an extension of the GRADE approach, which has previously been used in the determination of intervention effects for LiST [15]. While these analyses informed this process, the final effect sizes used in LiST often involved updates, adaptations, or adjustments based on expert consultation, context-specific considerations, or gaps in the evidence, rather than automatic and direct adoption of pooled estimates. We also reported the rationale and criteria used to update any existing effect sizes in LiST (as of 2024) and to introduce new interventions to the model. In addition, we have included some potentially effective and promising ‘innovative’ interventions for which the new evidence was currently insufficient to warrant inclusion within the standard LiST model. We provided a summary of the considerations for intervention recommendation and inclusion in LiST, adapted from the guidelines described by Walker *et al.* [15] (Table S1 in the **Online Supplementary Document**).

We used RevMan, version 5 (Cochrane, London, UK) for all updated meta-analyses.

## RESULTS

We organised the modelled interventions included in the three main packages of care by the timing of their delivery (**Table 1**; Table S2 in the **Online Supplementary Document**). We assessed several aspects of quality for each intervention, including outcome-level evidence quality (based on a GRADE assessment) (**Table 2**), as well as intervention effects on key outcomes and the specific cause of neonatal mortality to be modelled (**Table 3**). As LiST requires these cause-specific mortality estimates as inputs, we indicate which causes of death each intervention is linked to, and whether the chosen estimate is derived from a meta-analysis of a cause-specific mortality outcome, or an all-cause or unspecified mortality outcome where the major cause(s) are inferred by the specific disease targeted by an intervention (*e.g.* sepsis mortality affected by antibiotics). Rationale is provided for cases in which an intervention was reviewed but whose effect estimate remained unchanged, and cases in which interventions were not reviewed in the original comprehensive evidence synthesis and whose estimate was also unchanged are indicated.

**Table 1:** LiST packages of postnatal neonatal interventions by critical window

	Basic package (community/primary/secondary hospital care)	Referral care package (tertiary hospital care)	Innovation package
Immediate neonatal (first day of life)	Neonatal resuscitation (training), immediate drying and additional stimulation, clean birth environment, clean cord care (chlorhexidine), iKMC (facility), thermal protection*, nCPAP + preterm supportive care†, age-appropriate breastfeeding practices due to promotion*	Animal-derived surfactant*	Magnesium sulphate for neuroprotection in asphyxiated newborns†
Neonatal (second day to first week of life)	Community KMC, oral antibiotics, injectable antibiotics (secondary)	Prophylactic antifungals, injectable antibiotics (tertiary)†	Topical emollient therapy† for preterm, probiotics/synbiotics* for hospitalised preterm newborns
Cross-cutting (all critical windows)	Newborn referral and transportation†	Antibiotic stewardship: multicomponent strategies to reduce antimicrobial resistance, including regulation, education, and optimisation†	

iKMC – immediate kangaroo mother care, KMC – kangaroo mother care. LiST – Lives Saved Tool, nCPAP – nasal continuous positive airway pressure

*Intervention effects maintained from existing LiST model (no new review or update done).

†Intervention newly added to LiST.

**Table 2:** Summary of the quality of the evidence for postnatal newborn interventions

	Outcome	Study (participant) count	Study design(s)	Limitations (overall risk of bias)	Consistency (*I*^2^), %	Generalisability	Overall quality of evidence (GRADE)
**Immediate newborn interventions (first hour to first day of life)**							
Any neonatal resuscitation training *vs.* no neonatal resuscitation training with intervention-control data	Mortality due to asphyxia	3 (3,931)*	1 RCT, 2 cRCT	1 low risk, 1 some risk, 1 high risk	0	China, Zambia, Uganda	Low (wide CI, small event size, risk of bias concerns)
Clean birth environment (CBKs)	All-cause neonatal mortality	2 (23,932)	2 cRCT	1 some concerns, 1 high risk	0	2 Pakistan	Moderate (risk of bias from inability to blind participants and personnel)
Clean cord care (chlorhexidine) in community settings	All-cause neonatal mortality	5 (112,067)	1 RCT, 4 cRCT	3 low risk, 1 some concerns, 1 high risk	72	1 Bangladesh, 1 Nepal, 1 Tanzania, 1 Zambia, 1 Pakistan	Low (risk of bias concerns and inconsistency)
Clean cord care (chlorhexidine) in community settings	All-cause neonatal mortality	3 (54,624)	3 cRCT	2 low risk, 1 high risk	50	1 Bangladesh, 1 Nepal, 1 Pakistan (subset of studies with community births in high neonatal mortality settings)	Moderate (moderate to substantial heterogeneity)
iKMC (facility-based)	Mortality by 28 days	3 (3,533)	3 RCT	3 high risk	0	1 The Gambia, 1 Madagascar, 1 multicentre (Ghana, India, Malawi, Nigeria, and Tanzania)	High
nCPAP with supportive care for prematurity	Neonatal mortality	5 (322)	5 RCTs	2 low risk, 1 some risk, 2 high risk (blinding considered low risk for this outcome)	0	Tanzania, USA, UK, Australia (Historical studies)	Moderate (3 studies conducted in the pre-surfactant era, 4 small studies, not downgraded due to blinding - mortality outcome)
Animal-derived surfactant	Neonatal mortality	10 (1,469)	10 RCTs	NR	0	Finland, France, Germany, Italy, Japan, Netherlands, Sweden, Switzerland, Taiwan, UK, USA (Historical studies)	NR
Magnesium sulphate for neuroprotection	Neonatal mortality	13 (989)	13 RCTs	7 low, 3 some risk, 3 high risk	0	Bangladesh, Egypt, India, Pakistan, multi-centre (Malaysia, EMR region)	Moderate (some studies had high risk of bias)
**Neonatal interventions (second day to first week of life)**							
KMC continued in community	Neonatal mortality	4 (9,422)	RCTs	4 high risk	0	1 Ethiopia, 2 India, 1 Colombia	Low (due to risk of bias concerns)
Oral antibiotics in community settings for possible serious bacterial infections	All-cause neonatal mortality	2 (42,552)	2 cRCT	1 low risk, 1 high risk	0	1 Bangladesh, 1 Pakistan	Moderate (due to risk of bias concerns)
Injectable antibiotics (secondary and tertiary)	Neonatal mortality due to sepsis	13 (1,902)	13 RCTs	NA	NA	1 Malawi, 2 Kenya, 4 India, 1 Egypt, 1 Turkey, 1 Argentina, 2 Costa Rica, 1 multi-country (Estonia, Greece, Lithuania, Turkey, Italy, Spain)	NA – outcome data used to estimate survival rates after intervention
Injectable antibiotics (secondary and tertiary)	Neonatal mortality due to pneumonia	5 (770)	5 RCTs	NA	NA	4 China, 1 India	NA – outcome data used to estimate survival rates after intervention
Prophylactic systemic antifungals	All-cause neonatal mortality before hospital discharge	11 (1,551)	11 RCTs	1 low risk, 8 some concerns, 2 high risk	0	2 India, 1 Indonesia, 1 Iran, 1 Italy, 1 South Korea, 1 Turkey, 4 USA	Moderate (due to risk of bias concerns)
Topical emollient therapy for preterm neonates	All-cause neonatal mortality	10 (1,602)	10 RCTs	10 unclear risk	17	1 Bangladesh, 1 Brazil, 1 Egypt, 4 India, 1 Iran, 1 Pakistan, 1 Turkey	Low (serious limitations in study design leading to unclear risk of bias)
Probiotics or synbiotics for hospitalised preterm newborns	All-cause neonatal mortality	34 (7,488)	1 QE, 33 RCT	8 unclear risk, 8 low risk, 2 moderate risk, 16 high risk	0	1 Bangladesh, 1 Brazil, 1 Colombia, 2 China, 10 India, 1 Indonesia, 4 Iran, 2 Mexico, 1 Nepal, 1 Pakistan, 2 South Africa, 1 Thailand, 7 Turkey	Low (serious limitations in study design leading to high risk of selection, performance, and detection biases)
**Cross-cutting interventions (continued care for multiple critical windows)**							
Strategies to reduce antimicrobial resistance (regulation, education, and optimisation)	All-cause neonatal mortality	8 (28,928)	8 QE	7 moderate risk, 1 serious risk	85	3 China, 1 Egypt, 3 India, 1 Philippines	Very low (non-randomised studies, high heterogeneity, and risk of bias concerns)
Newborn referral and transportation	Neonatal mortality	1 (32,860)	1 cRCT	1 serious risk	NA	1 India	Very low (lack of blinding, only one study analysed)

CBK – clean birth kit, CI – confidence interval, cRCT – cluster randomised controlled trial, CS – cross-sectional study, iKMC – immediate kangaroo mother care, KMC – kangaroo mother care, NA – not applicable, nCPAP – nasal continuous positive airway pressure, NR – not reported, RCT – randomised controlled trial, QE – quasi-experimental trial

*Adjusted for clustering.

**Table 3:** Effects of key postnatal interventions to support newborn survival included in LiST

Intervention		Outcome	Previous LiST effect estimate	Updated/new LiST effect estimate
**Immediate newborn interventions (first hour to first day of life)**				
Neonatal resuscitation training programme, any training *vs*. no training	Neonatal mortality due to asphyxia	RR = 0.70 (95% CI = 0.59–0.84)	RR = 0.25 (95% CI = 0.11–0.59); intervention–control data	
	Neonatal mortality due to complications of prematurity	Facility births: Mdn = 10% reduction (IQR = 10–20); from Delphi	Original LiST estimate retained [8]	
Clean birth environment (clean birth kits) in facility settings		All-cause neonatal mortality	Only cause-specific neonatal mortality reported	RR = 0.73 (95% CI = 0.65–0.83)
		Neonatal mortality due to sepsis	27% reduction (IQR = 24–36%) from ‘clean birth practices in a facility’	Applied new all-cause neonatal mortality estimate – efficacy value unchanged
		Neonatal mortality due to tetanus	38% reduction (IQR = 34–40%) from ‘clean birth practices in a facility’	Original LiST estimate retained [16]
Clean cord care (chlorhexidine) in home or community settings		All-cause neonatal mortality	Only cause-specific neonatal mortality reported; Lancet ENAP 2014 [2] – RR = 0.77 (95% CI = 0.63, 0.94) – specific to umbilical antiseptics	Community births: RR = 0.77 (95% CI = 0.63–0.94; outcome not modelled directly; mixed community and facility births: RR = 0.88 (95% CI = 0.73–1.05; not modelled
		Neonatal mortality due to sepsis	40% (IQR = 25–50%) reduction from ‘clean newborn care practices at home during the postnatal period’	Applied new all-cause neonatal mortality estimate for trials focusing on home and community births in unclean settings
		Neonatal mortality due to tetanus	40% (IQR = 30–50%) reduction from ‘lean newborn care practices at home during the postnatal period’	Original LiST estimate retained [16]
iKMC (facility-based)		Neonatal mortality due to prematurity	RR = 0.75 (95% CI = 0.64–0.89) – 28 days; RR = 0.77 (95% CI = 0.58–1.04) – 72 hours	RR = 0.78 (95% CI = 0.66–0.92) – 28 days
Nasal continuous positive airway pressure *vs*. oxygen therapy (plus 10% additional effect for supportive care)		Neonatal mortality due to prematurity	Not in LiST; RR = 0.52 (95% CI = 0.32–0.87) (from Lancet ENAP 2014 [2])	RR = 0.53 (95% CI = 0.34–0.83); with +10% included: RR = 0.43 (95% CI = 0.24–0.73)
Animal-derived surfactant (including standard respiratory support)		All-cause neonatal mortality	Not in LiST; RR = 0.68 (95% CI = 0.57–0.82); from Lancet ENAP 2014 [2]; same source review	RR = 0.68 (95% CI 0.57–0.82); outcome not modelled directly
		Neonatal mortality due to prematurity	Not in LiST; Package in LiST: Full supportive care for prematurity – 80% reduction)	Applied all-cause neonatal mortality estimate
Magnesium sulphate for neuroprotection in asphyxiated infants*		Neonatal mortality due to asphyxia	Not in LiST	RR 0.83, 95% CI 0.69, 0.99
**Neonatal interventions (second day to first week of life)**				
KMC continued in community		Neonatal mortality due to prematurity	RR = 0.49 (95% CI = 0.29–0.82)	RR = 0.66 (95% CI = 0.51–0.85); initiated KMC within the first week of life
Oral antibiotics for possible serious bacterial infections		All-cause neonatal mortality	RR = 0.75 (95% CI = 0.64–0.89)	RR = 0.75 (95% CI = 0.69–0.82); outcome not modelled directly
		Neonatal mortality due to sepsis	28% reduction (IQR = 20–36.25%) reduction in sepsis-specific mortality (from Delphi)	Applied new all-cause neonatal mortality estimate
		Neonatal mortality due to pneumonia	RR = 0.58 (95% CI = 0.41–0.82)	Original LiST estimate retained
Injectable antibiotics (secondary) plus inpatient supportive care		Neonatal mortality due to sepsis	Injectable antibiotics for neonatal sepsis/pneumonia: 65% (IQR = 50-70%) from Delphi	80% (IQR = 0.75–0.85) reduction estimate based on observed survival rates and expert judgement
		Neonatal mortality due to pneumonia	Injectable antibiotics for neonatal sepsis/pneumonia: 75% (IQR = 70–81%) from Delphi	90% (IQR = 0.89–0.95) reduction estimate based on observed survival rates and expert judgement
Injectable antibiotics (tertiary) plus inpatient supportive care		Neonatal mortality due to sepsis	Full supportive care for neonatal sepsis/pneumonia: 80% (IQR = 75–85%) from Delphi	90% (95% CI = 0.88–0.91) reduction estimate based on observed survival rates and expert judgement
		Neonatal mortality due to pneumonia	Full supportive care for neonatal sepsis/pneumonia: 90% (IQR = 89-95%) from Delphi	95% (IQR = 0.89–0.95) reduction estimate based on observed survival rates and expert judgement
Prophylactic systemic antifungals		All-cause neonatal mortality prior to hospital discharge	Not in LiST	RR = 0.78 (95% CI = 0.62–0.99); outcome not modelled directly
		Neonatal mortality due to prematurity	Not in LiST	Applied all-cause neonatal mortality estimate
Topical emollient therapy for preterm*		All-cause neonatal mortality	Not in LiST; estimate from Lancet ENAP 2014 [2] – RR = 0.73 (95% CI = 0.56–0.94)	RR = 0.81 (95% = CI 0.70–0.94); outcome not modelled directly
		Neonatal mortality due to sepsis	Not in LiST	Applied new all-cause neonatal mortality estimate
		Neonatal mortality due to pneumonia	Not in LiST	Applied new all-cause neonatal mortality estimate
Probiotics or synbiotics for hospitalised preterm newborns*		All-cause neonatal mortality	Not in LiST	RR = 0.71 (95% CI = 0.59–0.85); outcome not modelled directly
		Neonatal mortality due to sepsis	Not in LiST	Applied new all-cause neonatal mortality estimate
		Neonatal mortality due to pneumonia	Not in LiST	Applied new all-cause neonatal mortality estimate
**Cross-cutting interventions (continued care for multiple critical windows)**				
Antibiotic stewardship strategies to reduce antimicrobial resistance (regulation, education, and optimisation)		All-cause neonatal mortality	Not in LiST	RR = 0.73 (95% CI = 0.57–0.93); outcome not modelled directly
		Neonatal mortality due to sepsis	Not in LiST	Applied new all-cause neonatal mortality estimate
Newborn referral and transportation		All-cause neonatal mortality	Not in LiST	RR = 0.76 (95% CI = 0.66–0.88); outcome not modelled directly
		Neonatal mortality due to prematurity	Not in LiST	Applied new all-cause neonatal mortality estimate
		Neonatal mortality due to asphyxia	Not in LiST	Applied new all-cause neonatal mortality estimate

CI – confidence interval, ENAP – Every Newborn Action Plan, iKMC – immediate kangaroo mother care, IQR – interquartile range, KMC – kangaroo mother care, LiST – Lives Saved Tool, Mdn – median, RR – risk ratio

*Interventions considered separately as part of an expanded package of recent innovative interventions

### Immediate neonatal interventions (first hour and day of life)

#### Neonatal resuscitation

For neonatal resuscitation training programmes, effects from a meta-analysis of randomised controlled trials (RCTs) assessing the effectiveness of any training programme *vs.* a control group were included in LiST. Neonatal resuscitation training programmes significantly reduced the risk of mortality due to asphyxia by 75% (risk ratio (RR) = 0.25; 95% CI = 0.11–0.59) [10]. This estimate showed a large effect magnitude and potential for clinical impact, a large sample size of pooled estimates, and low heterogeneity (*I*^2^ = 0), derived from trials conducted in China, Uganda, and Zambia.

#### Clean birth environment: clean birth kits

We updated the evidence on clean birth kits (CBKs) [8,16], pooling mortality data from two large studies with 23,932 participants from Pakistan [17,18]. We found that providing CBKs to birth attendants at home or at a healthcare facility significantly reduced the risk of all-cause neonatal mortality by 27% (RR = 0.73; 95% CI = 0.65–0.83) compared to not providing CBKs. Constituent trials delivered CBKs which included sterilised disposable gloves, soap, gauze, cotton balls, antiseptic solution, an umbilical-cord clamp, and a surgical blade [17] and CBKs with integrated care components allowing for the detection and response to early danger signs using 4% chlorhexidine, sunflower oil emollient, a temperature indicator sticker, a heat reflective polyester blanket, and a reusable heat pack [18]. Because no trials reported cause-specific neonatal mortality, we applied the 27% effect size to neonatal mortality due to sepsis. The existing effect on neonatal mortality due to tetanus within the LiST was retained.

#### Clean cord care (chlorhexidine)

We analysed mortality by predominant setting(s) of intervention application, *i.e.* whether they were healthcare facility-based, community-based (*i.e.* home application), or intended for a mixed setting (*i.e.* applications initiated in a healthcare facility and continued in the community). A facility-based study of 140 newborns in India found that chlorhexidine cleansing of the umbilical cord had no significant effect on all-cause neonatal mortality (RR = 0.11; 95% CI = 0.01–2.03) [19] compared to dry cord care. Likewise, a study of 1031 newborns in Pakistan found that mixed-setting application of chlorhexidine cleansing also had no significant effect on all-cause neonatal mortality (RR = 0.90; 95% CI = 0.47–1.70) [20] compared to dry cord care. The use of chlorhexidine for umbilical cord cleansing in community-based settings reduced the risk of all-cause neonatal mortality by 12% (RR = 0.88; 95% CI = 0.73–1.05) [21–25] compared to dry cord care, but this effect did not reach statistical significance [8]. No studies reported on cause-specific neonatal mortality.

In consultation with the TAG, we opted to maintain the previous Every Newborn Action Plan 2014 estimate (RR = 0.77; 95% CI = 0.63–0.94) for sepsis-related neonatal mortality in the LiST [26], which had been derived from the three community-based trials conducted in Bangladesh [21], Nepal [22], and Pakistan [25]. These trials had a high prevalence of births in the community (>80%) compared to the African trials [23,24], which had a higher proportion of facility-based deliveries in more controlled and clean circumstances. Two of the constituent trials also had high prevalences of potentially harmful cord applications such as ash, mud, and dung (84% [22] and 98% [25]). As such, these were deemed to be a more appropriate set of studies to model this intervention’s effects in unclean home and community settings, as they were derived from high neonatal mortality settings (≥30 per 1000 live births) where omphalitis would be more common. This is consistent with the findings reported by Lee Him *et al.* [8], which pointed to a consistent beneficial effect of cord chlorhexidine application on omphalitis rates, which can rapidly progress to neonatal sepsis and death if left untreated. The 40% (interquartile range (IQR) = 30–50) reduction in the risk of tetanus-specific mortality was retained from the existing LiST model and the original Delphi-derived estimate [16].

#### Drying, stimulation, thermal protection, and breastfeeding promotion

Three additional newborn interventions currently included in the LiST model had reviews that were not updated; their existing effects were retained as-is: immediate drying and additional stimulation [27], thermal protection [2], and early and exclusive breastfeeding promotion [28] (Table S3 in the **Online Supplementary Document**).

#### Immediate kangaroo mother care in facilities

According to our re-analysis [6] of the LMIC-specific data from the most recent systematic review of kangaroo mother care (KMC) [29], we identified a single study [30] investigating the effect of KMC provided immediately after birth. Two more studies [31,32] from this review [29] compared the effect of early-initiated KMC *vs*. late-initiated KMC. Pooled estimates from these three studies showed that KMC initiated within the first 24 hours of life reduced the risk of neonatal mortality by discharge or at 28 days of life by 22% (RR = 0.78; 95% CI = 0.66–0.92). While cause-specific mortality was not reported, given the target population for immediate kangaroo mother care (iKMC), we assigned the effect to neonatal mortality due to prematurity. The World Health Organization iKMC study [30] accounted for almost 88% of the weight of this pooled effect estimate given its large sample size. Early-initiated KMC also promoted exclusive breastfeeding at discharge (RR = 1.12; 95% CI = 1.07–1.16) and reduced hypothermia at discharge or 28 days of life (RR = 0.74; 95% CI = 0.61–0.90) and nosocomial sepsis until 28 days of life (RR = 0.85; 95% CI = 0.76–0.96) compared to late-initiated KMC. There was no difference in their effect on mortality by six months of age, and on exclusive breastfeeding at four weeks and six months of age. Subgroup analysis could not be assessed for all outcomes, as there were insufficient studies.

#### Nasal continuous positive airway pressure with supportive care

The use of nasal continuous positive airway pressure (nCPAP) was found to significantly decrease neonatal mortality by 47% when compared to oxygen therapy alone in preterm newborns with respiratory distress syndrome (RR = 0.53; 95% CI = 0.34–0.83). This effect was retrieved from a Cochrane review [33] which included five studies (322 participants), one from an LMIC (Tanzania) and four from HICs (USA, UK, and Australia). We opted to include the HIC studies, assuming they were conducted in settings with comparable clinical capacity to the Tanzania study, supported by a lack of heterogeneity (*I*^2^ = 0%). Three out of four HIC studies were conducted in the 1970s and the pre-surfactant era, while the remaining study was conducted in six secondary-level nurseries in 2007. This intervention is part of our basic package of interventions which should be available and accessible to all newborns in secondary facilities, especially those requiring additional respiratory support. A 47% reduction in deaths due to prematurity in neonates 1,000–2,000 g (including the affected fraction of 28.2% occurring in this birth weight range) was included in the LiST model for the impact of nCPAP.

#### Animal-derived surfactant with respiratory support

A significant 32% reduction in neonatal mortality was found when surfactant therapy was provided, alongside standard respiratory support (*i.e.* assisted ventilation and/or supplementary oxygen), compared to no surfactant among preterm newborns with respiratory distress syndrome (RR = 0.68; 95% CI = 0.57–0.82). We used this 32% value as the LiST effect on neonatal mortality due to prematurity for neonates requiring advanced care. This intervention is part of our referral/tertiary package of interventions that should be available and accessible at higher-level facility settings to support severe and specialised cases. These estimates were extracted from a Cochrane review [34], which is the last review that investigated animal-derived surfactant (human amniotic fluid extract, calf lung surfactant extract, and modified bovine surfactant extract, porcine surfactant extract) *vs.* control treatment (intratracheal administration of normal saline or air placebo, or no treatment). All ten studies (1,469 participants) were historical and conducted between 1980 and the 1990s in HICs (Finland, France, Germany, Italy, Japan, Netherlands, Sweden, Switzerland, Taiwan, UK, and USA).

Historical studies were used to generate estimates due to limited evidence from LMICs and ethical limitations for possible nCPAP *vs.* no nCPAP and surfactant *vs.* no surfactant trials, respectively. These estimates are distinct from the previous LiST estimate for full supportive care for prematurity (efficacy = 0.80), which used a Delphi process with a combination of the following interventions: warmth (thermal care and KMC), feeding support (direct and exclusive breastfeeding, gastric feeding, intravenous fluids), infection prevention and management (clean delivery practices, hand-washing, injectable antibiotics), oxygen administration/therapy, jaundice case management (phototherapy and exchange transfusion), surfactant, and nCPAP and intermittent positive pressure ventilation [2]. While we separately investigated the impact of select interventions from this Delphi process, we retained the assumption that all newborns are also receiving a full secondary care package for hospital-based nursing and supportive care, and have added an extra 10% (*i.e.* 0.1) to the CPAP effect estimate within the model to account for this. The baseline 47% effect from nCPAP alone is likely an underestimation in settings with minimal existing secondary care coverage for preterm newborns, given that the control groups in the included trials would have also received oxygen therapy and standard preterm supportive care. Thus, for the secondary facility care package (*i.e.* iKMC, nCPAP, and supportive care) for preterm neonates <2,000 g, this sums to a total 79% reduction in the risk of mortality due to prematurity (*i.e.* 0.22 + 0.47 + 0.1) – a value similar to the original Delphi estimate for the previous aggregate LiST intervention package of ‘Full supportive care for prematurity’. This was applied to an affected fraction of 28.2%, representing the percentage of preterm deaths occurring in birth weights of 1,000–2,000 g.

### Neonatal (second day to first week of life)

#### KMC after birth with continuation in the community

Initiated after 24 hours of life, KMC significantly reduced small vulnerable newborns’ neonatal mortality by discharge, 28 days of life, or 40 weeks post-menstrual age (RR = 0.69; 95% CI = 0.52–0.91) compared to conventional care [7]. We had a narrow population (preterm) and KMC initiation time frame that fit within the LiST model for this intervention. Therefore, we did an additional subgroup analysis to include studies that best fit these criteria and considered prior efforts to generate the LiST estimates for continued KMC. While these studies generated evidence for immediate community KMC initiation within the first day of life, there was insufficient evidence to differentiate these effects from standard KMC. Continued KMC (initiated within the first week of life) reduced neonatal mortality by 34% in this population (RR = 0.66; 95% CI = 0.51–0.85), which is the estimate included in LiST for impact on neonatal mortality due to prematurity. This was applied to an affected fraction of 29.7%, which is the proportion of neonatal deaths due to prematurity in newborns <2000 g. Three studies [35–37] followed participants for at least six months and found that the KMC group had a 25% lower mortality (RR = 0.75; 95% CI = 0.61–0.92) at six months than the conventional care group. For neonatal morbidities, KMC was associated with a lower risk of severe infection/sepsis (RR = 0.86; 95% CI = 0.79–0.93) and hypothermia (RR = 0.33; 95% CI = 0.19–0.55). It also improved exclusive breastfeeding and infant development in small vulnerable newborns.

Based on a large RCT from India [36], community-initiated KMC significantly reduced small vulnerable newborns’ neonatal mortality both by discharge (RR = 0.71; 95% CI = 0.52–0.96) and by six months of follow-up (RR = 0.76; 95% CI = 0.61–0.95). It also reduced the risk of severe infection/sepsis at the latest follow-up (RR = 0.89; 95% CI = 0.82–0.97) compared with conventional care. However, there was no significant effect on preventing hypothermia. Community-initiated KMC also significantly improved exclusive breastfeeding at discharge or 28 days of life (RR = 1.54; 95% CI = 1.49–1.59) and at 1–3 months of follow-up (RR = 1.82; 95% CI = 1.72–1.93). Because of the nature of this outcome and its downstream effects on different causes of neonatal mortality, we retained the existing impacts in LiST of KMC on exclusive and continued breastfeeding rates, including country-specific preterm birth rates.

#### Oral antibiotics for suspected serious bacterial infections

To prevent community-based infections in newborns with possible serious bacterial infections when facility referral is not possible, home delivery of oral antibiotics significantly reduced the risk of all-cause neonatal mortality by 25% (RR = 0.75; 95% CI = 0.69–0.82) [8,38]. We used this pooled effect estimate to model the effect of oral antibiotics on neonatal mortality due to sepsis. While we also evaluated early and late neonatal mortality and sepsis-specific neonatal mortality, only one study reported these outcomes [39]. When considering the timing of deaths, the risks of early (*i.e.* birth to six completed days; RR = 0.76; 95% CI = 0.68–0.84) and late (*i.e.* 7–27 completed days after birth; RR = 0.76; 95% CI = 0.61–0.96) neonatal mortality were both reduced by 24% compared to a standard hospital referral. Oral antibiotics administered in the community setting reduced the risk of sepsis-specific neonatal mortality by 22% (RR = 0.78; 95% CI = 0.60–1.03) compared to a standard hospital referral, though this result did not reach statistical significance. As we could not generate an updated estimate for neonatal mortality due to pneumonia, we retained the Every Newborn Action Plan 2014 effect estimate (RR = 0.58; 95% CI = 0.41–0.82) based on an analysis of three studies [40].

#### Injectable antibiotics for suspected or proven serious bacterial infection

We sourced hospital-based trials from a review by North *et al.* [41] which compared different antibiotic regimens to determine mortality rates after antibiotic treatment of newborns with possible serious bacterial infections. We compared the mortality rates across all treatment arms to a hypothetical scenario of 100% mortality if no antibiotic treatment were provided and found a 10.41% (95% CI = 9.07–11.87) mortality rate for all studies that included one or more non-HICs. Therefore, the implied mortality reduction from injectable antibiotics for possible serious bacterial infections in newborns receiving care in LMIC hospitals was 89.59%. Based on these calculations, a prior Delphi exercise [40] and consultation with our TAG, we estimated that injectable antibiotics with associated inpatient supportive care can lead to a 90% (95% CI = 0.88–0.91) reduction in sepsis-related deaths in tertiary care facilities and an 80% (IQR = 0.75–0.85) reduction in sepsis-related deaths in secondary care facilities (with an assumption of reduced supportive care capacity and provision). The point estimate and upper and lower bounds for secondary care are now equivalent to the prior Delphi estimate for ‘hospital-based management’ [40].

#### Prophylactic antifungals

Prophylactic systemic antifungal agents reduced the risk of neonatal mortality before hospital discharge by 22% (RR = 0.78; 95% CI = 0.62–0.99) in very preterm and very low birthweight newborns compared to control or placebo [8,43]. Given the limited data available from LMICs, we leveraged HIC data to produce a combined estimate. We assigned this intervention to achieve a 22% reduction in deaths due to prematurity, given the study population of very preterm and low birthweight newborns. Through expert discussion, it was decided that this intervention would be applied to preterm neonates requiring tertiary care and to the estimated affected fraction of 5% of admitted infants that would be expected to have sepsis.

### Cross-cutting (all critical windows)

#### Newborn referral and transportation

Timely and appropriate recognition, referral, and transportation for at-risk mothers and newborns is an essential part of the health system, and a crucial component in addressing preventable causes of neonatal mortality. A systematic review of perinatal care regionalisation and facility-based interventions [44] identified one cluster RCT [45] of a transportation intervention involving 32,860 participants, which found a 24% reduction in neonatal mortality (RR = 0.76; 95% CI = 0.66–0.88). This intervention involved a multi-component household-to-hospital continuum-of-care model that improved the quality of emergency obstetric and newborn care and leveraged community mobilisation to improve preparedness, knowledge, and access to transport to higher levels of care. Given the urgency of rapid transport and immediate intrapartum and postnatal intervention for mothers at risk of birth asphyxia and preterm birth, this 24% reduction of all-cause neonatal mortality estimate was applied to both asphyxia-related and preterm-related neonatal mortality within the LiST model.

#### Strategies to reduce antimicrobial resistance

Strategies to reduce the development and spread of antimicrobial resistance are essential across all critical care windows. Implementing regulation and optimisation interventions did not significantly reduce excess mortality due to nosocomial bloodstream infections (RR = 0.62; 95% CI = 0.31–1.25) compared with standard practices or no intervention. Implementing multimodal strategies of regulation, education, and optimisation significantly reduced the risk of all-cause mortality by 27% (RR = 0.73; 95% CI = 0.57–0.93) compared with standard practices or no intervention [8]. Therefore, we selected the comprehensive effect of implementing all three antimicrobial resistance reduction strategy types in tertiary or higher referral facilities for modelling and applied it to the outcome of neonatal mortality due to sepsis. This effect is applied to the proportion of neonates surviving after antibiotic use for the treatment of infection (65%).

The quasi-experimental nature of the included studies reflects the ethical challenges of not assigning a randomised comparator group to receive basic training on infection prevention and control and appropriate, judicious antibiotic administration. The overall very low quality of the evidence and considerable statistical heterogeneity for all-cause neonatal mortality stem from the differences in the interventions implemented across the included studies, even though most were carried out in tertiary care facilities. Despite this, sensitivity analyses omitting high-risk-of-bias studies indicated the main analysis was robust and the direction of effect consistently favoured the post-intervention period, suggesting a true effect of mortality reduction.

### Recent innovations and promising interventions

The interventions presented within the below section were considered for inclusion within the LiST model as a separate ‘innovation package’ that was not part of the basic intervention package. These interventions still have developing evidence base and require more research to determine their potential for impact on neonatal mortality.

#### Magnesium sulphate for neuroprotection in neonates

For term neonates with hypoxic-ischaemic encephalopathy as a consequence of birth asphyxia or other intrapartum complications, 13 trials assessing magnesium sulphate, either as a sole neuroprotective therapy administered postnatally within one hour of birth or as an adjunct with therapeutic hypothermia, found it significantly reduced the risk of neonatal mortality before discharge by 17% (RR = 0.83; 95% CI = 0.69–0.99) [9,46]. Results on the composite outcome of death or neurological disabilities, however, did not reach significance. None of the other pharmacological agents (allopurinol, erythropoietin and melatonin) investigated for neuroprotection in hypoxic-ischaemic encephalopathy had a significant effect on newborn mortality. We therefore used a 17% reduction in the risk of neonatal mortality due to asphyxia for magnesium sulphate. We applied an affected fraction of 10% for the proportion of asphyxia mortality that is susceptible to its neuroprotective effects.

#### Topical emollients

For preterm newborns in facility settings, topical emollients reduced the risk of all-cause neonatal mortality by 19% (RR = 0.81; 95% CI = 0.70–0.94) compared to routine skin care [8,47]. To update the LiST model, we used this overall combined estimate of a 19% reduction in mortality due to both sepsis and pneumonia. Small differences emerged when assessing the effects subgrouped by emollient type: topical ointments or creams reduced neonatal mortality by 18% (RR = 0.82; 95% CI = 0.69–0.98), and topical oils reduced neonatal mortality by 22% (RR = 0.57; 95% CI = 0.61–1.00).

#### Probiotics and synbiotics

The combined effects of newborn supplementation with either probiotics or synbiotics showed a significant 29% reduction in the risk of all-cause neonatal mortality (RR = 0.71; 95% CI = 0.59–0.85) compared to control [8]. Individually, probiotics significantly reduced the risk of neonatal mortality by 25% (RR = 0.75; 95% CI = 0.61–0.92) in preterm newborns, and synbiotics significantly reduced the risk of neonatal mortality by 47% (RR = 0.53; 95% CI = 0.33–0.85) in preterm and low birthweight newborns compared to control. We used the combined effect of probiotic and synbiotic supplementation as a newly introduced intervention in the LiST model and applied the effect to neonatal mortality due to both sepsis and pneumonia. We assigned both topical emollients and probiotics/synbiotics to the fraction of hospital admissions with very low birth weight (*i.e.*<32 weeks’ gestation or <1.5 kg birth weight).

## DISCUSSION

We summarised evidence on the effects of postnatal interventions on cause-specific neonatal mortality from clinical trial data in existing, updated, and *de novo* systematic reviews and meta-analyses. Additionally, we presented the rationale for adding specific interventions and updating existing impact estimates based on more recently published trial data. This study follows the comprehensive evidence synthesis and associated lives saved modelling carried out as part of the 2014 Lancet Every Newborn Series [2].

Newly introduced interventions recommended for inclusion in the core package include nCPAP and associated preterm supportive care, animal-derived surfactant, prophylactic antifungals, newborn referral and transportation, and antibiotic stewardship and education. The previously aggregated intervention of ‘full supportive care for prematurity’ has been split into two components, including nCPAP (plus supportive care) at the secondary care level and animal-derived surfactant administration at the tertiary care level. Together with iKMC, these three interventions comprise the hospital-based supportive care package for preterm newborns weighing <2,000 g.

Effect sizes of several interventions changed in magnitude based on more recent experimental evidence relevant to LMICs (**Table 3**). For example, more recent research has shown neonatal resuscitation training programmes can be substantially more effective at reducing neonatal mortality. Including additional new trials led to a modest reduction in the point estimate for the impact of KMC continued in the community on neonatal mortality but also tightened the CI around the effect.

Several innovative and promising interventions that were introduced for separate consideration include magnesium sulphate for neuroprotection in asphyxiated newborns and topical emollient therapy and probiotics/synbiotics for hospitalised preterm newborns. During modelling for impact on lives saved, interventions can be combined into packages (*e.g.* basic package, level of facility care, innovative interventions) as appropriate to the context.

Several limitations should be addressed regarding the data sources for the intervention effect estimates. While an ideal scenario includes multiple robust, large RCTs contributing to the meta-analysed estimate, such data were not consistently available for mortality outcomes. Because of the nature of the LiST model, a key assumption was that all-cause mortality outcomes could be assigned to one or more specific causes of death.

In some cases (*e.g.* newborn referral and transportation), data from only one large trial was available. In other cases, a combination of data on neonatal survival rates from antibiotic drug comparison studies and pre-existing Delphi consensus estimates and ranges (*e.g.* injectable antibiotics at the secondary and tertiary levels), or historical data (*e.g.* CPAP and surfactant), was used where placebo-controlled studies were no longer feasible due to ethical concerns. In these cases, decisions were made through expert discussion to select the most robust available evidence on intervention effects and inform the development of a model incorporating a comprehensive package of maternal and neonatal interventions. However, for estimates requiring historical data from HICs, it is important to consider whether these data apply to, and are feasible within, LMICs today. Low-resource settings may have different health system capacities and reduced access to, and availability of, the infrastructure, healthcare personnel, and resources required for the interventions assessed. This is a key reason why LMIC-specific evidence was prioritised for inclusion and synthesised in the companion reviews. Future implementation in these settings requires careful consideration of the costs and contextual realities that may affect appropriate scaling and monitoring to ensure adequate newborn care.

Furthermore, intervention effect estimates represent only one aspect of the LiST model. Population-affected fractions also estimate the proportion of cause-specific mortality attributable to the condition targeted by each intervention, and these estimates are updated periodically [3]. Variation in the availability and reliability of intervention coverage data introduces additional uncertainty and may affect how accurately projected intervention scale-up translates into estimates of lives saved. To account for uncertainty in coverage at baseline and at scale-up targets, coverage ranges can be incorporated into the scenarios used for analysis.

The quality of delivered interventions also varies between and within countries, which may influence the extent to which reported coverage translates into lives saved. Ultimately, the LiST model provides a set of default modelling inputs and relies on critical assessment from individual users to adapt these modelling inputs and create a fit-for-purpose analysis for their decision-making needs. This involves consideration of intervention delivery quality, coverage, effects, and health system capacity.

## CONCLUSIONS

We summarised the most up-to-date evidence available for a set of postnatal newborn interventions. We synthesised the impact of a package of effective postnatal interventions for neonates to update the LiST model. The new interventions and updated effect estimates can provide more accurate projections of the potential impacts on neonatal survival of different intervention coverage scale-up scenarios in LMICs.

## Additional material


Online Supplementary Document

## Data Availability

**Data availability:** All secondary data used in this paper was collected from the articles cited herein and can be found in the original publications.
