# Supplementary material for: Postnatal interventions to support newborn survival: updates for the Lives Saved Tool
Source: J Glob Health. 2026 Oct 2;16:04272. doi: 10.7189/jogh.16.04272 (PMC13631769; doi:10.7189/jogh.16.04272)
Supplement: Supplementary file 1 [file jogh-16-04272-s001.pdf]

Supplement to: Vaivada T, Tam Y, Dominguez G, Jiang L, Lee Him R, Muralidharan O, Sihota D, Harrison L, Black RE, Bhutta ZA. Postnatal interventions to support newborn survival: updates for the Lives Saved Tool. J Glob Health. 2026;16:04272.

Table S1. Summary of possible considerations for recommendation of a newborn intervention for scale-up and inclusion in the LiST based on available evidence

	**Considerations, explanations, and examples**
**Outcome and pooled effect estimate**	
Magnitude and significance of effect estimate	The strength of the effect and potential for clinical relevance or impact at the population level
Nature of outcome	Is the outcome related to mortality (all-cause or cause-specific), morbidity, development, or disability?; Can the outcome be considered a risk factor (*e.g.* preterm birth), or is it a direct measurement of a clinical endpoint (*e.g.* mortality, cerebral palsy)?; If there is no measured or insufficient effect on a clinical endpoint, can a recommendation still be made when there is a strong effect on a key risk factor?
Width of 95% CI	Indicator of certainty of the effect estimate
Total sample size of pooled estimate	Indicator of reliability of the effect estimate
Total number of studies included in pooled estimate	A higher number of studies allows better assessment of potential consistency of effect across different settings and populations
**Included studies and trials**	
Design of included studies	More robust quality study designs (*e.g.* RCT) contribute to higher strength of evidence
Methodological quality and risk of bias of included studies	Lower assessed risk of bias contributes to higher strength of evidence
Heterogeneity of included studies	Indicator of the consistency of the effect across settings and populations
Results of subgroup and/or sensitivity analyses	Allows for the determination of effect differences between settings, populations, or other measurable characteristics of the intervention
**Applicability of evidence to LMICs**	
LMIC-specific evidence available	Provides evidence for effectiveness that is more aligned with recommendations for resource-constrained settings
Proportion of LMIC-specific evidence	Proportion of evidence that is more aligned with recommendations for resource-constrained settings
Potential application and feasibility of intervention in LMICs	Pragmatic assessment of the real-world potential for implementation and scale up of the intervention in resource-constrained settings
Ability to leverage, and applicability of, HIC evidence where LMIC data unavailable	Pragmatic assessment of the comparative burden of disease and expected variation in the actual effect of an intervention between HIC and LMIC settings

CI – confidence interval, HIC – high-income country, LiST – Lives Saved Tool, LMIC – low- and middle-income country, RCT – randomised controlled trial

Table S2. Names and definitions of postnatal interventions included in the Lives Saved Tool

Intervention Name	Definition	Package
IMMEDIATE NEONATAL CARE (FIRST DAY OF LIFE)		
Clean birth environment (including clean birth kits)	Percent of deliveries with clean/sterile environment from kit or infection prevention measures (i.e. handwashing, clean perineum, clean surface), including use of clean instrument to cut and tie the umbilical cord.	Basic
Neonatal resuscitation training	Percent of deliveries that require opening the airway and ventilating newborn babies who do not spontaneously cry/breathe after birth.	Basic
Immediate drying and additional stimulation	Percent of deliveries where healthcare provider thoroughly dries and stimulates the newborn by rubbing the back 2-3 times.	Basic
Clean cord care with chlorhexidine in home or community settings	Percent of deliveries where application of antiseptics (chlorhexidine) to cord after cutting was performed in home or community delivery settings.	Basic
iKMC (facility-based)	Percent of prematurely born neonates who have access to and receive hospital-based full supportive care plus immediate kangaroo mother care.	Basic
Thermal protection	Percent of deliveries for which drying of the baby immediately after birth, skin-to-skin contact with mother, wrapping, and no bath in first 6 hours was performed.	Basic
nCPAP with supportive care for prematurity	Percent of prematurely born neonates who have access to and receive hospital-based respiratory support, including nasal continuous positive airway pressure and supportive care.	Basic
Age-appropriate breastfeeding practices due to promotion	Percentage of children whose mothers receive activities designed to promote breastfeeding. Breastfeeding promotion can either be one-on-one or group meetings.	Basic
Animal-derived surfactant (including standard respiratory support)	Percent of prematurely born neonates who have access to and receive full respiratory care including ventilation and surfactant therapy at a tertiary or referral facility.	Referral
Magnesium sulfate for neuroprotection in asphyxiated infants	Percent of neonates with birth asphyxia that receive magnesium sulfate as part of post-asphyxial aftercare.	Innovation
EARLY NEONATAL CARE (SECOND DAY TO FIRST WEEK OF LIFE)		
Kangaroo Mother Care (KMC) continued in community	Percent of premature neonates receiving Kangaroo Mother Care (KMC). KMC is defined as continuous skin-to-skin contact between a mother and her newborn as well as frequent and exclusive breastfeeding.	Basic
Oral antibiotics in community settings for	Percent of children with suspected pneumonia (symptoms of acute respiratory infec on) for whom advice or treatment was sought from a health facility or provider.	Basic

possible serious bacterial infections		
Injectable antibiotics (secondary facility) plus inpatient supportive care	Percent of neonates with suspected sepsis/pneumonia treated with injectable antibiotics and receive inpatient supportive care in secondary facility settings	Basic
Prophylactic systemic antifungals	Percent of preterm neonates admitted with suspected sepsis who receive prophylactic antifungals	Referral
Injectable antibiotics (tertiary facility) plus inpatient supportive care	Percent of neonates with suspected sepsis/pneumonia treated with injectable antibiotics and receive inpatient supportive care in tertiary or referral facility settings.	Referral
Topical emollient therapy for preterm	Percent of prematurely born neonates who receive topically applied emollients to support skin barrier integrity for the prevention of infection.	Innovation
Probiotics or synbiotics for hospitalized preterm newborns	Percent of hospitalized prematurely born neonates who receive probiotic or synbiotics.	Innovation
CROSS-CUTTING (ALL CRITICAL WINDOWS)		
Newborn referral and transportation	Percent of at-risk mothers and neonates who have access to and receive transportation to referral services to prevent and mitigate complications of birth asphyxia or preterm birth.	Basic
Antibiotic stewardship strategies to reduce antimicrobial resistance (multi-component strategies including regulation, education, and optimization)	Percent of neonates with suspected sepsis/pneumonia being treated within a hospital where antibiotic stewardship interventions have been successfully implemented.	Referral
iKMC = immediate kangaroo mother care; nCPAP = nasal continuous positive airway pressure;		

Table S3. Additional newborn interventions existing in the LiST model

Intervention	Outcome	Effect Estimates
**Immediate drying and additional stimulation**	Neonatal mortality due to asphyxia	10% reduction (IQR 5–15% from Delphi)
	Neonatal mortality due to prematurity	10% reduction (IQR 5–15% from Delphi)
**Thermal protection**	Neonatal mortality due to prematurity	20% reduction (IQR 15–25% from Delphi)
	Neonatal mortality due to sepsis	10% reduction (IQR 8–17% from Delphi)
**Age-appropriate breastfeeding practices due to promotion**	Early initiation of breastfeeding and exclusive breastfeeding	Various direct impacts on early initiation of breastfeeding and exclusive breastfeeding, with downstream impacts on neonatal infectious disease-associated mortality
